# The Effect of Conditioned Media of Adipose-Derived Stem Cells on Wound Healing after Ablative Fractional Carbon Dioxide Laser Resurfacing

**DOI:** 10.1155/2013/519126

**Published:** 2013-12-04

**Authors:** Bing-Rong Zhou, Yang Xu, Shi-Lei Guo, Yan Xu, Ying Wang, Fen Zhu, Felicia Permatasari, Di Wu, Zhi-Qiang Yin, Dan Luo

**Affiliations:** ^1^Department of Dermatology, The First Affiliated Hospital of Nanjing Medical University, Nanjing 210029, China; ^2^Nanjing Regenerative Medicine Engineering and Technology Research Center, Nanjing 210029, China

## Abstract

*Objective.* To evaluate the benefits of conditioned medium of Adipose-derived stem cells (ADSC-CM) on wound healing after fractional carbon dioxide laser resurfacing (FxCR) on human skin. *Materials and Methods.* Nineteen subjects were treated with FxCR on the bilateral inner arms. ADSC-CM was applied on FxCR site of one randomly selected arm. Transepidermal water loss (TEWL), skin color, and gross-elasticity of FxCR site on both arms were measured. Skin samples were taken by biopsy from three subjects 3 weeks after treatment for histopathological manifestations and mRNA expressions of procollagen types I and III, elastin genes were noted. *Results.* The index of erythema, melanin, and TEWL of the ADSC-CM-treated skin were significantly lower than those of the control side. The mRNA expression of type III procollagen in ADSC-CM-treated group at 3 weeks posttreatment was 2.6 times of that of the control group. *Conclusion.* Application of allograft ADSC-CM is an effective method for enhancing wound healing after FxCR, by reducing transient adverse effects such as erythema, hyperpigmentation, and increased TEWL.

## 1. Introduction

The concept of fractional photothermolysis has opened a new era of laser resurfacing by providing the ability to obtain significant clinical results with minimal posttreatment recovery [1]. Fractionated technology has led to the development of a number of nonablative and ablative devices. Reports on the nonablative fractionated devices emphasized nearly complete absence of prolonged side effects. On the contrary, ablative fractionated devices, such as fractional carbon dioxide (CO_2_) resurfacing (FxCR), provide a treatment method that makes dozens of small wounds on the skin surface, called microthermal treatment zones, and transmits thermal injury into skin dermis, thus reforming collagen [2]. Reports on FxCR have shown that clinical complications can occur, such as skin swelling, postinflammatory hyperpigmentation, prolonged erythema, and scarring [2–7]. Given that some of these complications are potentially avoidable, new modalities to accelerate the wound healing after FxCR have recently become a new research focus.

Adipose-derived stem cells (ADSCs), a kind of mesenchymal stem cells within the stromal-vascular fraction of subcutaneous adipose tissue, display multilineage developmental potentiality and secrete various growth factors influencing the damaged neighboring cells [8–10]. Evidences have been accumulated that demonstrate the wound healing effects of ADSCs [8, 11–14]. Kim et al. first demonstrated that ADSCs accelerate wound healing in *ex vivo* and *in vivo* experiments [8]. They also observed that the conditioned media of ADSC (ADSC-CM), which stimulated the migration of dermal fibroblasts after wounds, were made in primary cultured fibroblasts [8]. ADSCs secreted a variety of growth factors such as basic fibroblast growth factor (bFGF), keratinocyte growth factor (KGF), transforming growth factor (TGF)-*β*, hepatocyte growth factor (HGF), and vascular endothelial growth factor (VEGF) into the conditioned medium, which might have mediated the wound-healing effect of ADSCs [15]. In addition to the *in vitro* evidence, the wound healing effect of ADSC-CM was also verified in an animal study, which showed that topical administration of ADSC-CM significantly reduced the wound size and accelerated the reepithelialization at the wound edge [8, 15]. Therefore, ADSCs and their soluble factors are promising for wound healing and antiphotoaging therapy [16, 17]. The purpose of this study is to evaluate the effectiveness and benefits of ADSC-CM on wound healing after FxCR on human skin.

## 2. Materials and Methods

### 2.1. Isolation and Culture of ADSCs

This study was approved by the Institutional Review Board of the First Affiliated Hospital of Nanjing Medical University. Allograft ADSC-CM was prepared as previously described [18]. Human subcutaneous adipose tissue samples were obtained from two selective liposuctions of healthy HIV/HBV/HCV-negative females with informed consent. The obtained samples were digested with 0.075% collagenase type II (Sigma-Aldrich, St. Louis, MO) under gentle agitation for 45 minutes at 37°C and centrifuged at 300 ×g for 10 minutes to obtain the stromal cell fraction. The pellet was filtered with a 70 mm nylon mesh filter and resuspended in phosphate-buffered saline. The cell suspension was layered onto histopaque-1077 (Sigma-Aldrich Company Ltd., Poole, UK) and centrifuged at 840 ×g for 10 minutes. The supernatant was discarded, and the cell band buoyant over histopaque was collected. The retrieved cell fraction was cultured overnight at 37°C/5% CO_2_ in culture medium (endotoxin ≤10 EU/mL) (Dulbecco's modified Eagle's media (DMEM; HyClone, Logan, UT, USA), 10% fetal bovine serum (FBS; Gibco BRL, Gaithersburg, MD, USA), 100 U/mL of penicillin, and 100 mg/mL of streptomycin (Beyotime, Jiangsu, China)). The resulting cell population was maintained over 3–5 days until confluence, which were represented as in passage 1. ADSCs were cultured and expanded in culture medium and used for the experiments (passage 3).

### 2.2. Identification of DAs

The phenotype of ADSCs (passage 3) was evaluated by flow cytometry analysis (FACS, BD Biosciences, San Jose, CA) by using PE-labeled anti-human CD29, FITC-labeled anti-human CD34, FITC-labeled anti-human CD71, and PE-labeled anti-human CD90 monoclonal antibodies (Chemicon, USA). PE- and FITC-conjugated mouse monoclonal antibodies (Chemicon, USA) of irrelevant specificity were tested as negative controls. To induce osteogenic and adipogenic differentiation, ADSCs (passage 3) were grown to approximately 90% confluence; then the medium was replaced into adipogenic or osteogenic differentiation medium. The adipogenic medium was complete medium supplemented with 1.0 *μ*M dexamethasone, 10 mg/mL insulin, 100 *μ*M indomethacin, and 500 *μ*M IBMX (Sigma-Aldrich, St. Louis, MO). The osteogenic medium was supplemented with 50 *μ*M ascorbic acid, 0.1 *μ*M dexamethasone, and 10 mM *β*-glycerophosphate (Sigma-Aldrich, St. Louis, MO). The induced cells were cultured for up to 20 days with medium exchange every three days, and then the differentiated ADSCs were stained with Oil Red O (Sigma-Aldrich, St. Louis, MO) for detection of lipid droplet in adipogenic induction or Alizarin Red (Sigma-Aldrich, St. Louis, MO) for calcium in osteogenic induction.

### 2.3. Collection of ADSC-CM

When the ADSCs (passage 3) reached confluence, the medium was changed into an FBS free DMEM medium (StemPro MSC SFM XenoFree medium, Life Technologies, Gaithersburg, MD). After changing the medium, ADSCs were exposed to hypoxia (2% O_2_, 5% CO_2_, and balanced N_2_) for 72 hours. Then, conditioned media of ADSCs were collected, centrifuged at 300 ×g for 5 minutes, and finally filtered using a 0.22 mm syringe filter.

### 2.4. Detection of Several Cytokines in ADSC-CM by ELISA

The concentrations of several cytokines involving wound healing in ADSC-CM were measured using sandwich ELISA kits according to the manufacturer's instructions; VEGF, TGF-*β*1, bFGF, KGF, platelet-derived growth factor (PDGF)-A, and HGF ELISA kits were obtained from R&D Systems (Minneapolis, MN). All the experiments were performed in duplicate.

### 2.5. Clinical Study Protocol

Nineteen healthy Chinese volunteers (five men and fourteen women), whose ages ranged from 24 to 33 years old with Fitzpatrick skin types III-IV, were enrolled in the study after completing informed consent. We excluded subjects who had a history of FxCR treatments or cosmetic procedure on the inner arms in the last 12 months, with skin lesions on the inner arms or with any bleeding tendency. The bilateral inner arms, were treated by a fractional CO_2_ laser (Crius, Han's Laser, Shenzhen, China). Each side was irradiated at 8 and 16 mJ with the same spot density (30%), giving a total of four treatment sites over both arms. We used the dosage of 8 mJ and 16 mJ in the present study because these dosages represent the two most commonly used dosages (high dosage and low dosage) in our clinical practice with this fractional CO_2_ laser apparatus. The area of each treatment site was 1 cm^2^.

ADSC-CM was topically applied onto FxCR-treated sites of one randomly selected arm for one hour, while FBS free DMEM medium was applied to FxCR-treated sites of the other arm. The dermatological changes: the index of erythema, melanin, TEWL, and elasticity were measured with respective probes: TEWAmeter, Mexameter, and Cutometer (Courage & Khazaka Electronic GmbH, Cologne, Germany) as instructed on days 1, 4, 7, 14, and 21 after laser treatment. Clinical photographs were also taken on days 1, 4, 7, and 14.

For histopathological analysis, biopsy samples were taken from both arms of three subjects on day 21. Serial sections (4 mm) were mounted onto silane-coated slides and stained by H&E, Masson-Trichrome, and Gomori's aldehyde fuchsin staining, respectively.

The mRNA expressions of procollagen types I and III, elastin genes were determined according to the manufacturer's protocol (KeyGen Biotech Co., Ltd., Nanjing, Jiangsu, China). Total RNA was extracted from biopsy skin samples by using TRIzol (Invitrogen, USA). cDNA was synthesized from the isolated RNA using SuperScript III Reverse Transcriptase (Keygen, China). PCR was performed on ABI Prism 7700 Sequence Detector (Applied Biosystems). Specific primers were listed in Table 1. For data analysis, the ^ΔΔ^Ct method was used. For each gene, fold-change was calculated as the difference in gene expression between two groups. A positive value indicated gene upregulation and a negative value indicated gene downregulation. The results were expressed as mean ± SD of 3 independent experiments.

### 2.6. Statistical Analysis

The results were analyzed with the paired *t*-test using SPSS 15.0 software (SPSS, Inc., Chicago, IL). *P* < 0.05 was considered to be significant.

## 3. Results

**Figure 1:**
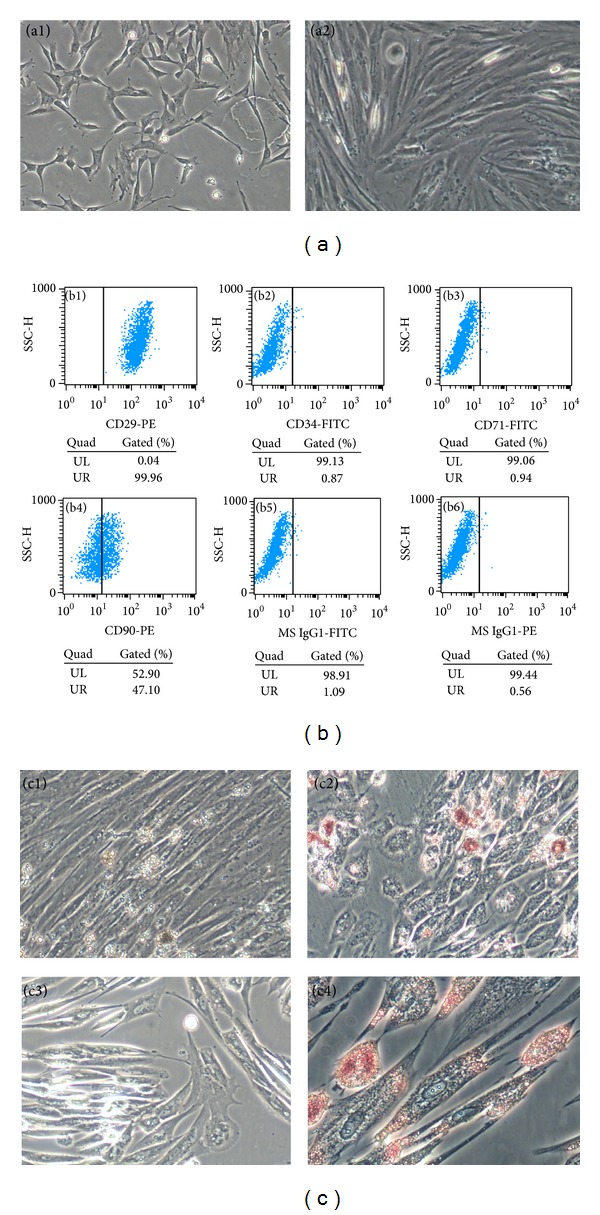
Characterization of adipose stem cell. The primary adipose stem cells exhibited a typical fibroblast-like morphology (a1) and were harvested at passage 3 (a2) for flow cytometry analysis. The results confirmed that the cells express CD29 (99.96%, b1), CD90 (47.1%, b4), CD34 (0.87%, b2), and CD71 (0.94%, b3). PE (0.56%, b5) and FITC (1.09%, b6) were performed as negative control. Oil Red O staining of lipid after differentiation for 28 days (before staining, c1; after staining, c2) and Alizarin Red staining of calcium after differentiation (before staining, c3; after staining, c4).

To characterize the ADSCs population, the morphological and phenotypic properties of ADSCs were investigated using light microscopy and FACS, respectively. Purified adipose stem cells were harvested at passage 3 (Figure 1(a)). Flow cytometry analysis of ADSCs stained with anti-CD29, anti-CD34, anti-CD71, and anti-CD90 confirmed that ADSCs expressed CD29 (Figure 1(b1)) and CD90 (Figure 1(b4)) and were lacking in CD34 (Figure 1(b2)) and CD71 (Figure 1(b3)). Adipogenesis, induced by a long time exposure to adipogenic medium, was monitored and confirmed based on the appearance of lipid vacuoles by Oil Red O staining (Figure 1(c2)). Meanwhile, from osteogenic differentiation, Alizarin Red staining obviously revealed that those ADSCs could deposit calcium, as seen from a red staining (Figure 1(c4)). TGF-*β*1, VEGF, bFGF, KGF, PDGF, and HGF was detected in ADSC-CM used in the present study by ELISA method. As listed in Table 2, the concentration of TGF-*β*1, VEGF, bFGF, KGF, PDGF-A, and HGF were 126.76 ± 2.71 pg/mL, 136.04 ± 29.23 pg/mL, 65.09 ± 18.47 pg/mL, 91.43 ± 10.41 pg/mL, 48.12 ± 1.33 pg/mL, and 648.43 ± 47.29 pg/mL, respectively.

Four groups of data were compared: FxCR 8 mJ with ADSC-CM (T1), FxCR 8 mJ with DMEM medium (C1), Fx CR 16 mJ with ADSC-CM (T2), and FxCR 16 mJ with DMEM medium (C2). The average baseline parameters showed no statistical differences among the groups.

**Figure 2:**
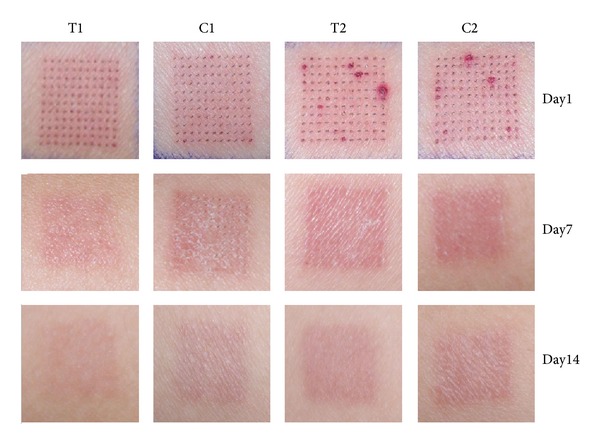
Representative clinical photography of fractional carbon dioxide laser-treated sites. The ADSC-CM-treated side shows less erythema and hyperpigmentation. FxCR 8 mJ with ADSC-CM (T1), FxCR 8 mJ with DMEM medium (C1), FxCR 16 mJ with ADSC-CM (T2), and FxCR 16 mJ with DMEM medium (C2).

**Figure 3:**
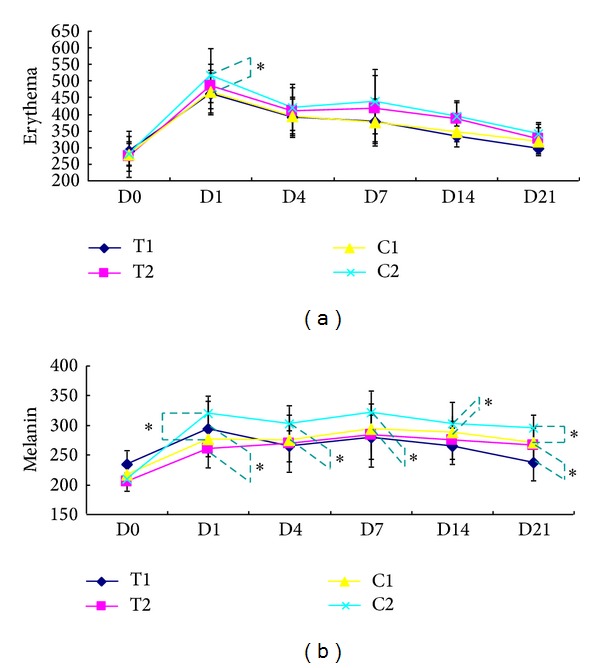
(a) Erythema index (EI) (b) and melanin index (MI) after fractional carbon dioxide (CO_2_) laser resurfacing. T1 and T2 are the average values of the indices in the ADSC-CM-treated sites after a single pass of 8 and 16 mJ, respectively. C1 and C2 are the average values in the DMEM applied sites after a single pass of 8 and 16 mJ, respectively. D0: before treatment; D1: 1 day after treatment; D3: 3 days after treatment; D7: 7 days after treatment; D14: 14 days after treatment; and D21: 21 days after treatment.*P < 0.05 with the paired *t*-test.

Figure 2 shows representative clinical manifestation of 4 groups taken at indicated time points. The ADSC-CM-treated side (both T1 and T2) shows less erythema and less pigmentation, especially at days 7 and 14. The intensity of skin redness was measured using the erythema index (EI). During the study, the average value of EI decreased continuously after achieving the peak value at day 1. The ADSC-CM-treated side showed a significant difference of lower EI than that of the 16 mJ treated sites at day 1 (*P* < 0.05) (Figure 3(a)). The melanin index (MI) represents the darkness of the skin. Similar to the tendency of EI, the average value of MI reached its peak at day 1 and then decreased gradually until the end of the study. The ADSC-CM-treated side had a lower average value of MI, and statistically significant difference was observed between T2 and C2 at days 1, 7, 14, and 21 (*P* < 0.05). Interestingly, the MI value is significantly higher in T1 than that of C1 at day 1. However, the MI value decreased gradually in the following days and showed a significantly lower MI level than that of C1 at day 21 (*P* < 0.05).

**Figure 4:**
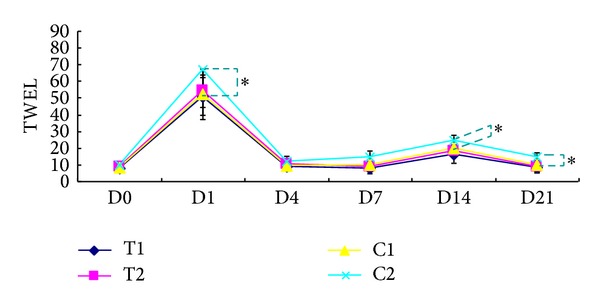
Transepidermal water loss (TEWL) after fractional carbon dioxide (CO_2_) laser resurfacing. T1 and T2 are ADSC-CM-treated sites after a single pass of fractionated CO_2_ laser energy of 8 and 16 mJ, respectively. C1 and C2 are the DMEM-applied control sites after single passes of 8 and 16 mJ, respectively. D0: before treatment; D1: 1 day after treatment; D3: 3 days after treatment; D7: 7 days after treatment; D14: 14 days after treatment; and D21: 21 days after treatment. *P < 0.05 with the paired *t*-test.

The value of TEWL reached its peak at day 1 after FxCR treatment and then rapidly decreased to nearly baseline at day 4 (Figure 4). A slight reincrease in TEWL was observed at approximately day 7–14; then TEWL decreased again at day 21. This transient reincrease in TEWL was thought to be caused by exfoliation of the crust. All four groups showed a similar change pattern in TEWL. In all subjects, the ADSC-CM-treated side showed a greater reduction of TEWL than the control side in the 16 mJ treated group at days 1, 14, and 21 (Figure 4).

**Figure 5:**
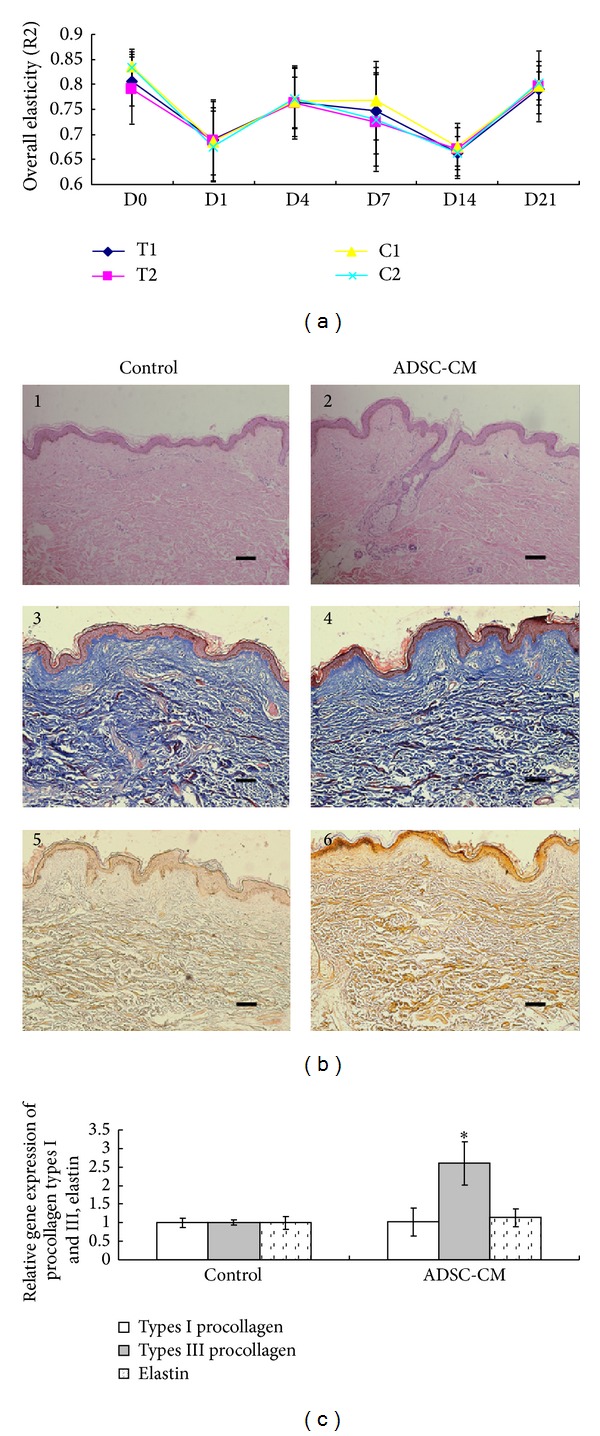
(a) Overall elastin (R2) after fractional carbon dioxide (CO_2_) laser resurfacing. T1 and T2 are ADSC-CM-treated sites after a single pass of fractionated CO_2_ laser energy of 8 and 16 mJ, respectively. C1 and C2 are the DMEM-applied control sites after single passes of 8 and 16 mJ, respectively. D0: before treatment; D1: 1 day after treatment; D3: 3 days after treatment; D7: 7 days after treatment; D14: 14 days after treatment; and D21: 21 days after treatment. (b) HE (1 and 2), Masson-Trichrome (3 and 4), and Gomori's aldehyde fuchsin (5 and 6) staining of biopsy specimen from fractional carbon dioxide laser-treated sites on day 21. Scale bar 50 *μ*m. (c) Total RNA was extracted from biopsied skin samples of different groups. Procollagen types I and III and elastin mRNA were determined by real-time RT-PCR analysis. Results are shown as means ± SD (*n* = 3). The asterisk (∗) indicates a significant difference (*P* < 0.05) between the control group and the ADSC-CM-treated group.

R2, the main parameter to assess the skin elasticity, is represented by the ratio of redeformation ability of skin to final distension and is referred to as the gross-elasticity of the skin [19]. As shown in Figure 5(a), R2 decreased to a relatively lower level at day 1–14. This transient decrease in R2 was thought to be caused by edema and formation of crusts on the skin. R2 value recovered to baseline level in all four groups at day 21. However, no significant differences were observed between ADSC-CM-treated side and DMEM-treated side at indicated time points. Representative HE, Masson-Trichrome, and Gomori's aldehyde fuchsin staining images of biopsy samples obtained from control and ADSC-CM-treated side on day 21 posttreatment are shown in Figure 5(b). Histopathologic examination of specimens from the bilateral inner arms of two subjects showed no significant changes in H&E staining, including epidermis, dermis, and skin appendages. No significant changes could be quantified in elastin stain between ADSC-CM-treated sides and the control. Although type I procollagen and elastin mRNA between ADSC-CM-treated and control skin samples showed no significant differences, the mRNA of type III procollagen in ADSC-CM-treated group was 2.6 times that of control group. Adverse effects such as infection, prolonged erythema, and scarring were not observed at either side in any group.

## 4. Discussion

ADSCs are multipotent cells that have the ability to differentiate along multiple lineages giving rise to bone cartilage or fat. The cultured ADSCs of this study could differentiate into adipogenic and osteogenic lineages in specific culture medium. Our cultured ADSCs showed the same immunophenotypes as previously described [20–22]. They expressed the mesenchymal stem cell markers CD29 and CD90 but not the hematopoietic marker CD34.

Recently, autologous ADSCs were found to accelerate wound healing in human cutaneous wounds. Kim et al. first demonstrated that ADSCs accelerated skin wound healing [8]. Besides, the wound healing effect of ADSCs was also verified in animal studies, which showed that topical administration of ADSCs significantly reduced the wound size [15, 23, 24]. Further studies showed that ADSCs promote proliferation of dermal fibroblasts, not only by direct cell-to-cell contact, but also by paracrine cytokines. Notably, as shown by present and other researchers' results (Table 2), a variety of growth factors can be secreted by ADSCs into the conditioned medium, including TGF-*β*1, VEGF, bFGF, KGF, PDGF-A, and HGF [8, 9, 17, 25], which contribute to major wound healing effect of ADSCs. Compared with ADSCs, there are some clinical application advantages of ADSC-CM. (i) In clinical practice, allogenic ADSCs cannot be directly applied to the skin of patients. However, there are no cells in ADSC-CM; thus, the immunoreactions can be avoided when using ADSC-CM. (ii) In order to harvest autologous ADSCs, patient should undergo a selective liposuction operation. Allogenic ADSC-CM can be used without the need for patients to undergo surgical operations. (iii) ADSC-CM can be prepared during ADSCs culture as they have plenty of rich sources. These benefit the clinical usage of ADSC-CM over ADSCs. The culture medium of DMEM and FBS used in the present study was of GMP grade and free from endotoxin. The safety of the topical ADSC-CM in this study can also be guaranteed. Our present results demonstrated for the first time that the acceleration of the healing process after FxCR could be achieved by topical application of ADSC-CM.

The ADSC-CM-treated side showed less erythema in all subjects. Even though ADSC-CM contains some paracrine cytokines related to angiogenesis, such as VEGF [26, 27], no obvious angiogenesis was observed in our study. By contrast, the erythema caused by FxCR was significantly reduced by topical ADSC-CM application, indicating that it can prevent the common complications of FxCR. The exact underlying mechanism by which ADSC-CM could reduce erythema remains to be elicited; nevertheless, ADSC-CM is helpful to achieve optimum clinical outcomes after FxCR skin rejuvenation.

We also found in our study that ADSC-CM reduced transient hyperpigmentation after FxCR. The antioxidant activity of the whitening growth factors in ADSC-CM has been elucidated to account for the whitening effect of ADSCs [28, 29] by inhibiting the synthesis of melanin and the activity of tyrosinase in a dose-dependent manner and downregulating the expression of melanogenic enzymes, such as tyrosinase-related protein-1 and TGF-*β*1 [28]. Generally, the increased level of TEWL indicates impaired skin barrier function. In our study, the changes of TEWL showed that ADSC-CM could fasten recovery of the skin barrier function, which could be explained as ADSC-CM activated the proliferation and migration of human primary keratinocytes as reported [30]. Postinflammatory hyperpigmentation is thought to be caused by phagocytozing of the impaired basal keratinocytes and melanocytes by melanophages, which contain a large amount of melanin and accumulate in the upper dermis. The rehabilitation of TEWL by ADSC-CM may be another explanation for reduced transient hyperpigmentation after FxCR treatment.

From the findings of skin elasticity measurement and histopathological results, there was no obvious changed elasticity or increased collagen at ADSC-CM-treated sites. It was reported that ADSC-CM could increase the protein expression of type I collagen and decrease that of MMP-1 in fibroblasts, which may explain the increased collagen content of the dermis in animal experiments [17, 25, 31]. Fractional carbon dioxide laser has already been proven to be an effective transdermal drug-delivery system both *in vitro* and *in vivo*, which may also be helpful to facilitate delivery of ADSC-CM to dermis [32]. In our real-time RT-PCR results, although no obvious changes of type I procollagen and elastin mRNA expression were found, we did find type III procollagen to be increased in ADSC-CM-treated samples. The predominant type of collagen in the dermis is type I, followed by small amounts of type III. Type I collagen is characterized by thick fibers that confer stiffness and resistance to perform a crucial function in maintaining the structure of dermis, whereas type III collagen is characterized by thin fibers that present the resiliency of skin [33]. Theoretically, from others' and our results, ADSC-CM might enhance the skin rejuvenation effect of FxCR; however, the skin tightening effect was not observed in our 3-week study even though type III procollagen mRNA increased. This may be due to the limited concentration of the ADSC-CM cytokines *in vitro* and the limited observation period. Based on this consideration, further investigation on the skin rejuvenation effect of ADSC-CM is still needed.

In summary, the results of the present study strongly suggest that the application of ADSC-CM could be effective for enhancing wound healing after FxCR skin rejuvenation, by reducing transient adverse effects. Since this is just a small pilot study, more randomized controlled clinical trials with large samples are needed to prove it, including studies with patients of more skin types and multiple treatment parameters. The treatment in this study was conducted on off-facial sites, whereas most laser resurfacing is performed on the face, so the wound healing may be different. Overall, our results suggest for the first time that topical ADSC-CM application may enhance the recovery of wound healing after laser resurfacing, which deserves further investigation.

## 5. Conclusion

The results of the present study strongly suggest that the topical application of allogenic ADSC-CM could be an effective method for enhancing wound healing and reducing transient unwanted adverse effects after FxCR skin rejuvenation.

## Figures and Tables

**Table 1:** Primers used in the real-time RT-PCR amplification of the human types I and III procollagen, elastin, and GAPDH mRNAs.

Gene name	Primer sequences
Type I procollagen	
Forward primer	5′-AGGACAAGAGGCATGTCTGGTT-3′
Reverse primer	5′-TTGCAGTGGTAGGTGATGTTCTG-3′

Type III procollagen	
Forward primer	5′-TGGATCAGATGGTCTTCCA-3′
Reverse primer	5′-TCTCCATAATACGGGGCAA-3′

Elastin	
Forward primer	5′-GCT AAG GCA GCC AAG TAT GG-3′
Reverse primer	5′-CAG CTC CAA CCC CGT AAG TA-3′

GAPDH	
Forward primer	5′-TGTTGCCATCAATGACCCCTT-3′
Reverse primer	5′-CTCCACGACGTACTCAGCG-3′

**Table 2:** Concentration of several cytokines in conditioned media of ADSCs.

Cytokines	Concentration (pg/mL)
TGF-*β*1	126.76 ± 2.71
VEGF	136.04 ± 29.23
bFGF	65.09 ± 18.47
KGF	91.43 ± 10.41
PDGF-A	48.12 ± 1.33
HGF	648.43 ± 47.29
